# CG-RecNet: a gated and attention-fused deep learning framework for label-free classification of neural stem cell differentiation via imaging flow cytometry

**DOI:** 10.3389/fcell.2026.1767574

**Published:** 2026-02-16

**Authors:** Qinzi Li, Fang Liu, Junyu Zhou, Xuanjian Zou, Chenlin Gao, Jingze Li

**Affiliations:** 1 College of Life Sciences, Sichuan Agricultural University, Ya’an, China; 2 College of Water Resources and Hydropower, Sichuan Agricultural University, Ya’an, China; 3 College of Science, Sichuan Agricultural University, Ya’an, China; 4 College of Information Engineering, Sichuan Agricultural University, Ya’an, China

**Keywords:** attention mechanism, deep learning, explainable AI, high-throughput screening, label-free classification, neural stem cells

## Abstract

**Introduction:**

Precise and longitudinal monitoring of Neural Stem Cell (NSC) differentiation is pivotal for advancing regenerative medicine. However, traditional identification methods rely on invasive immunochemical staining, which terminates cell viability and precludes real-time analysis.

**Methods:**

To address these limitations, we propose CG-RecNet, a specialized deep learning framework for accurate, label-free classification of NSC differentiation lineages—specifically neurons, astrocytes, and oligodendrocytes—directly from brightfield imaging flow cytometry (IFC) data. The architecture integrates a LinAngular Cross-Channel Attention (LinAngular-XCA) Fusion Module to capture global morphological dependencies and a Gated Convolutional Neural Network (GatedCNN) Block to suppress background noise.

**Results:**

Validation on rat embryonic NSCs indicates that CG-RecNet achieves an overall accuracy of 96.40% and a macro-average AUC of 0.9979, representing a 1.82% improvement over established baselines. Notably, the model achieved high precision in identifying the minority oligodendrocyte lineage without synthetic oversampling.

**Discussion:**

Grad-CAM analysis indicates that the model’s attention aligns with biologically relevant hallmarks, such as neurite outgrowth and soma texture. CG-RecNet provides a reliable, non-invasive, and qualitatively interpretable tool for neural stem cell research.

## 1 Introduction

Neurological disorders, encompassing acute traumatic injuries and chronic neurodegenerative conditions such as Alzheimer’s disease (AD), Parkinson’s disease (PD) (Kalia and Lang, 2015), and Multiple Sclerosis (MS), constitute a profound global health burden (Feigin et al., 2019). The pathophysiology of these conditions is complex, typically characterized by neuronal loss, pathogenic protein accumulation, and widespread demyelination (Jucker and Walker, 2018; Hauser and Oksenberg, 2006; Long and Holtzman, 2019). Crucially, the Central Nervous System (CNS) response to such pathology is heavily mediated by glial cells. While astrocytes and oligodendrocytes play indispensable roles in maintaining homeostasis and facilitating signal conduction, their dysregulation—manifesting as reactive astrogliosis or inflammatory crosstalk—can significantly impede neural regeneration (Linnerbauer et al., 2020; Sofroniew, 2009; Siracusa et al., 2019). For instance, the glial scar, while containing injury, often acts as a physical barrier to axonal regrowth (Bradbury and Burnside, 2019; Fawcett and Asher, 1999; Cieri and Ramos, 2025), whereas the failure of oligodendrocyte precursor cells to remyelinate axons marks the functional decline in MS (Franklin et al., 2024; Warnock et al., 2020).

Given this context, Neural Stem Cells (NSCs) have emerged as a pivotal therapeutic strategy due to their intrinsic capacity for self-renewal and multipotent differentiation into neurons and glia (Li et al., 2024; Dimou and Götz, 2014). The transplantation of exogenous NSCs or the mobilization of endogenous progenitors holds significant promise for replacing lost neurons and modulating the immune microenvironment (De Gioia et al., 2020; Martino et al., 2011). Consequently, identifying and directing the fate of NSCs—specifically distinguishing between functional neurons and supporting glial phenotypes—is critical for the efficacy of cell-based therapies (Gao et al., 2023). However, tracking NSC fate *in vivo* and *in vitro* remains a significant challenge (Xue et al., 2022). Traditional identification relies heavily on molecular assays such as immunofluorescent staining. Although these techniques provide specific molecular markers, they are inherently invasive and destructive, precluding the real-time, longitudinal monitoring of live cell cultures required for high-throughput drug screening.

To overcome these methodological bottlenecks, artificial intelligence has been increasingly integrated into biomedical research. Deep learning (DL), particularly Convolutional Neural Networks (CNNs), has demonstrated substantial potential in medical image analysis (Jia et al., 2024), automating complex diagnostic tasks with high precision (Litjens et al., 2017; Esteva et al., 2017; Zhou et al., 2021). In the realm of cellular imaging, DL algorithms have successfully segmented cells, analyzed phenotypes, and performed “*in silico* labeling”—predicting fluorescent labels from label-free brightfield images (Krikid et al., 2024; Mao and He, 2024; Moen et al., 2019; Christiansen et al., 2018).

Building on these advancements, Zhu et al. pioneered a deep learning-based approach specifically for NSC differentiation (Zhu et al., 2021). Their work established that label-free brightfield microscopy images contain sufficient morphological information to distinguish between neuronal and glial lineages without chemical staining. This validated the feasibility of using deep learning as a non-invasive alternative to biological assays. However, current state-of-the-art approaches primarily rely on standard CNN backbones such as ResNet (He et al., 2016) or Xception. While effective at extracting local texture features, these architectures employ fixed receptive fields that often struggle to capture long-range morphological dependencies—such as the correlation between the soma and distant neurite extensions—which are essential for distinguishing subtle phenotypes (Vaswani et al., 2017; Fujitani et al., 2017). Furthermore, standard convolutions lack explicit mechanisms to suppress the background noise and cellular debris inherent in label-free brightfield imaging, potentially compromising classification accuracy in complex culture environments (Yang et al., 2025).

To address these limitations, we propose CG-RecNet, a specialized deep learning framework engineered to enhance the predictive precision of NSC differentiation using high-throughput imaging flow cytometry. Built upon a ResNet50 backbone, our architecture integrates a LinAngular Cross-Channel Attention (LinAngular-XCA) Fusion Module (Zhou et al., 2023b) to explicitly model global semantic dependencies and a Gated Convolutional Block to robustly suppress background noise while refining local feature extraction. While IFC simplifies the requirement for cell segmentation compared to tissue microscopy, the challenge remains in distinguishing subtle, fine-grained phenotypic differences in low-contrast brightfield images. By synergistically processing both global context and local texture details, our model aims to overcome the inductive bias limitations of conventional CNNs. We comprehensively validate our approach on established datasets and provide interpretability via heatmap visualizations, offering a reliable and transparent tool for accelerating NSC research and therapeutic development.

## 2 Proposed methodology

### 2.1 Overview of the proposed framework

**FIGURE 1:**
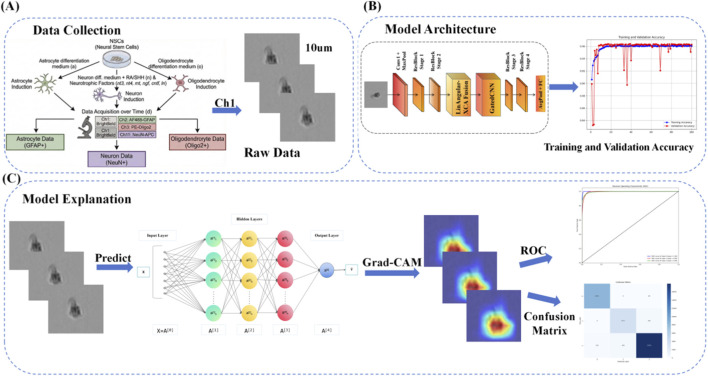
Workflow of the CG-RecNet framework. **(A)** Data Collection: Acquisition of brightfield NSC images. **(B)** Model Architecture: ResNet50 augmented with LinAngular-XCA and GatedCNN. **(C)** Model Explanation: Grad-CAM interpretability and quantitative evaluation.

The workflow of the CG-RecNet system is illustrated in Figure 1. The framework consists of four stages: (1) Data Acquisition, where brightfield images are collected during NSC differentiation; (2) Preprocessing, including geometric transformations and normalization to enhance model generalization; (3) Model Training, where the CG-RecNet architecture—leveraging ResNet50-based feature extraction, LinAngular-XCA, and Gated CNNs—classifies the three neural cell types; and (4) Interpretation, employing Grad-CAM to visualize decision regions.

### 2.2 Data Acquisition and class definition

The experimental dataset utilized in this study is derived from the “Deep learning-based predictive identification of neural stem cell differentiation” database, constructed by Zhu et al. and publicly available on the Figshare platform. This dataset comprises a substantial collection of single-cell images acquired via high-throughput imaging flow cytometry, with samples originating from primary Neural Stem Cells (NSCs) derived from embryonic Sprague-Dawley (SD) rats.

The categorization of samples into three distinct classes—astrocytes (Class 0), oligodendrocytes (Class 1), and neurons (Class 2)—reflects a classification logic based on a lineage-specific differentiation strategy, rather than mere morphological clustering. This classification paradigm aligns with the fundamental principles of developmental neurobiology, wherein pluripotent NSCs undergo directed differentiation toward specific fates guided by distinct environmental cues. For instance, as detailed in Table 1, neurons (Class 2) are defined through specific induction utilizing agents such as Retinoic Acid (RA) and Sonic Hedgehog (SHH); this category constitutes the largest subset (124,403 samples). In contrast, the glial lineage is represented by astrocytes (55,466 samples) and oligodendrocytes (27,687 samples), which were induced by their respective differentiation media. Although this strategy results in class imbalance, we explicitly retained this original distribution to evaluate the model’s capacity to identify subtle, biologically dependent feature representations defined by the source benchmark under varying sample densities.

**TABLE 1:** Dataset composition and label mapping.

Class id	Proposed label (English)	Original folder name (Portuguese)	Sample count
0	Astrocytes	NSCs treated with astrocyte differentiation medium	55,466
1	Oligodendrocytes	NSCs treated with oligodendrocyte differentiation medium	27,687
2	Neurons	NSCs treated with neuron differentiation medium (with retinoic acid (RA) and sonic hedgehog (SHH),etc.)	124,403

**FIGURE 2:**
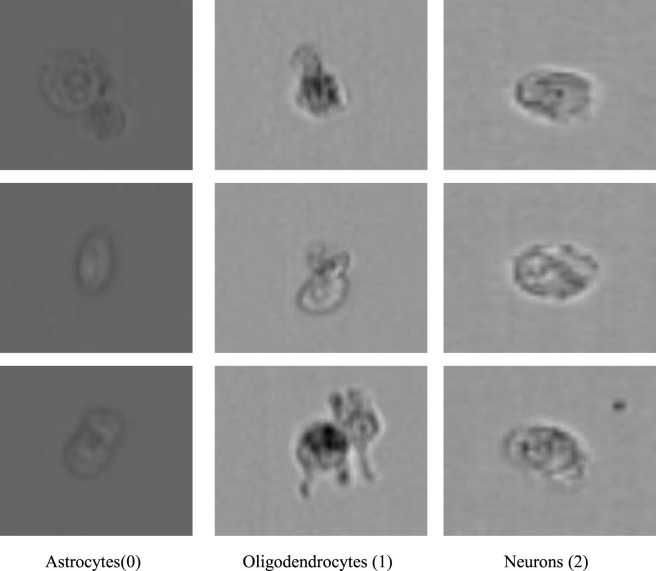
Visualization of representative samples from the dataset. The images are arranged sequentially from 0 to 2, corresponding to the three categories of neural cells: Astrocytes (0), Oligodendrocytes (1), and Neurons (2). All images were acquired using the Brightfield channel (Ch1) of the imaging flow cytometer. Scale bar = 10 μm.

**FIGURE 3:**
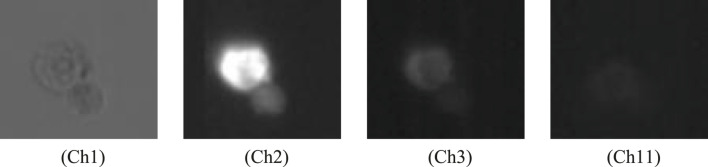
Representative multi-channel images acquired via imaging flow cytometry. The figure displays the label-free Brightfield channel (Ch1) alongside the corresponding fluorescence channels used as biological ground truth references: Ch2 (AF488-GFAP), Ch3 (PE-Oligo2), and Ch11 (NeuN-APC). Scale bar = 10 μm.

To facilitate the development of a non-invasive classification framework, this study utilized images from the Brightfield channel (Ch1). While the source database included corresponding fluorescence channels—AF488-GFAP, PE-Oligo2, and NeuN-APC—which established the biological Ground Truth, processing in this study was conducted based on the brightfield modality to evaluate classification performance relying on intrinsic morphological features. For data integrity, the original folder structure provided by the dataset curators was maintained. Table 1 details the correspondence among the proposed Class IDs, the directory names for specific induction treatments, and the sample size for each category. Figure 2 provides a visualization of representative brightfield images for each of the three categories. Additionally, Figure 3 illustrates representative multi-channel images, displaying the Brightfield channel (Ch1) alongside the fluorescence channels (Ch2, Ch3, and Ch11) used as reference.

### 2.3 Data preprocessing

To ensure optimal input quality for the deep learning framework and to enhance model robustness against cellular morphological variations, this study implemented a systematic preprocessing pipeline applied to all raw brightfield single-cell images. This pipeline comprises three key stages: geometric standardization, statistical normalization, and random data augmentation.

First, to standardize the spatial dimensions required by the ResNet50 backbone, we implemented a consistent resizing strategy. For the validation and test sets, images were first resized to 256 pixels along the short edge, followed by a Center Crop to extract a unified 224 × 224 pixel region of interest (ROI). This deterministic processing ensures that evaluation metrics reflect the model’s recognition performance on the most salient cellular features without introducing artificial geometric distortions.

Subsequently, to facilitate stable convergence during gradient descent optimization, pixel intensity values were converted into floating-point tensors within the range [0, 1]. Next, channel-wise Z-score normalization was applied using ImageNet dataset statistics (mean μ = [0.485, 0.456, 0.406] and standard deviation σ = [0.229, 0.224, 0.225]). This step standardizes the input distribution, aligning it with the distribution of the backbone network’s pretrained weights.

Finally, to mitigate overfitting and improve generalization capability regarding different cellular orientations, data augmentation was performed exclusively on the training dataset. The augmentation strategy incorporated Random Resized Cropping, which randomly samples crop regions from the original images and resizes them to 224 × 224 pixels, thereby simulating variations in cellular scale and imaging focus. Additionally, random horizontal flipping with a probability of 0.5 was applied to accommodate the rotational invariance inherent in suspension cell imaging (Shorten and Khoshgoftaar, 2019). Regarding dataset gating, raw IFC data were pre-gated based on area and aspect ratio to remove debris and doublets, ensuring that the majority of inputs represented single cells. However, minor physical aggregates may remain, reflecting real-world high-throughput screening conditions. Crucially, to strictly prevent data leakage and ensure an unbiased evaluation of the model’s diagnostic capabilities, no random augmentation techniques were applied to the validation or test sets; they were strictly maintained in a standardized, deterministic state (Varma and Simon, 2006).

Algorithm 1 **Preprocessing and Augmentation Pipeline for Neural Cell Images.**

**Input:** Raw brightfield single-cell dataset $D=\left\{\left(I_{i},y_{i}\right)\right\}$

**Output:** Augmented Training Set $D_{\text{train}}^{\prime }$ , Standardized Validation Set $D_{\text{val}}^{\prime }$ , and Test Set $D_{\text{test}}^{\prime }$

**Begin**
 Split D into $D_{\text{train}}$ , $D_{\text{val}}$ , $D_{\text{test}}$

**Function** Preprocess(Image I ):
 
$I\leftarrow \text{Resize}\left(I,256\right)$

 
$I\leftarrow \text{CenterCrop}\left(I,224\times 224\right)$

 
$I\leftarrow \text{ToTensor}\left(I\right)/225.0$

 
$I\leftarrow \text{Normalize}\left(I,\mu =\left[0.485,\ldots \right],\sigma =\left[0.229,\ldots \right]\right)$

 
$I\leftarrow \text{RandomHorizontalFlip}\left(I,p=0.5\right)$

**Return** I

**End Function**
**For** each image $I_{i}$ in $D_{\text{val}},D_{\text{test}}$ **do**:
 
$I_{i}^{\prime }\leftarrow \text{StandarPreprocess}\left(I_{i}\right)$

**End For**
Apply stochastic augmentation for generalization
**For** each image $I_{j}$ in $D_{\text{train}}$ **do**:
Apply stochastic augmentation for generalization
 
$I_{j}^{\prime }\leftarrow \text{TrainAugment}\left(I_{j}\right)$

Add processed sample to $D_{\text{train}}^{\prime }$

**End For**
**Return** $D_{\text{train}}^{\prime },D_{\text{val}}^{\prime }{,D}_{\text{test}}^{\prime }$

**End**

### 2.4 Proposed deep learning architecture and model details

**FIGURE 4:**
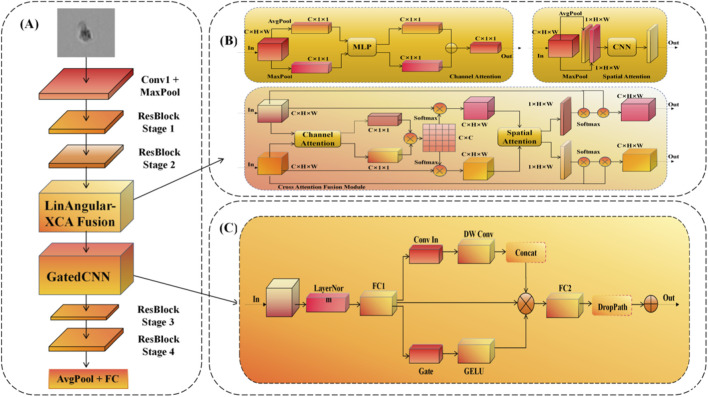
Overview of the Proposed Model Architecture. **(A)** The CG-RecNet framework, augmenting ResNet50 with LinAngular-XCA Fusion and Gated CNN Block. **(B)** LinAngular-XCA Fusion Module for global morphological modeling. **(C)** Gated CNN Block for noise suppression and local feature refinement.

To address the inherent limitations of standard Convolutional Neural Networks (CNNs) in capturing the multiscale morphological intricacies of differentiating Neural Stem Cells (NSCs), we propose a unified framework termed CG-RecNet. While traditional backbones such as ResNet50 excel at extracting hierarchical features, they rely heavily on local convolutions with fixed receptive fields, which often limits their ability to model global dependencies and effectively suppress background noise in label-free microscopy. As illustrated in Figure 4, our framework advances the ResNet50 backbone by integrating two novel architectural components strategically placed after the second residual stage: the LinAngular Cross-Channel Attention (LinAngular-XCA) Fusion Module and the Gated Convolutional Neural Network (GatedCNN) Block (Dauphin et al., 2017; Liu et al., 2022). Specifically, the LinAngular-XCA module is designed by synergistically integrating Linear-complexity Angular Attention (Zhou et al., 2023b) and Cross-Covariance Attention (XCA) (El-Nouby et al., 2021) through a cross-fusion framework inspired by recent hybrid attention architectures (Zhou et al., 2023a). This strategic placement ensures that the model captures both high-resolution spatial features and long-range semantic dependencies early in the feature extraction process.

To rigorously evaluate the contribution of each component, we define two intermediate model variants: ResCMNet, which incorporates only the attention fusion mechanism to enhance global context, and ResGDNet, which utilizes only the gated convolution to refine feature selection. The final CG-RecNet synergistically combines both modules to achieve reliable lineage prediction.

#### 2.4.1 LinAngular-XCA Fusion Module (ResCMNet)

The accurate discrimination of cell fates relies on distinguishing subtle membrane textures and capturing long-range morphological dependencies, such as the correlation between nuclear elongation and distant neurite outgrowth. Standard convolutions, limited by local receptive fields, often fail to model these global interactions. To address this, we introduce the LinAngular-XCA Fusion Module (Figure 4B). Inspired by the dual-branch paradigm of the Convolutional Block Attention Module (CBAM) (Woo et al., 2018), our design advances this concept by integrating two specialized mechanisms—LinAngular Attention for spatial dependencies and Cross-Covariance Attention for channel interactions—via a cross-fusion strategy (Zhou et al., 2023a).

To capture global spatial contexts without the quadratic computational complexity ( $O\left(N^{2}\right)$ ) of standard self-attention, we incorporate the LinAngular Attention mechanism. Unlike conventional dot-product attention, this component exploits the associativity of matrix multiplication to achieve linear complexity with respect to sequence length. The input feature map X is projected into query ( Q ), key ( K ), and value ( V ) embeddings. To ensure numerical stability and consistent feature magnitude during the linear approximation, we introduce specific normalization terms. The computation is formally expressed as Equation 1:

$$\text{LinAngular}\left(X\right)=N\left(\frac{1}{\pi }Q\left(K^{T}V\right)+0.5V\right),$$ (1)

where 1/π serves as an empirical angular scaling factor to stabilize gradient flow, N denotes Layer Normalization, and the computation order $\left(K^{⊤}V\right)$ reduces the complexity to linear scale.

Complementary to spatial processing, we incorporate a Cross-Covariance Attention (XCA) branch to explicitly model global interactions between feature channels (El-Nouby et al., 2021). This process generates a global covariance map by applying attention operations along the channel dimension rather than the spatial dimension, defined as Equation 2:

$$\text{XCA}\left(Q,K,V\right)=V\cdot \text{Softmax}\left(\frac{Q^{T}K}{\tau }\right),$$ (2)

where τ is a learnable temperature parameter that scales the inner product, effectively highlighting co-activated feature channels associated with specific lineage markers.

Distinct from approaches that apply these maps sequentially, we implement a Cross-Attention Fusion strategy adapted from Zhou et al. (2023a). This submodule projects refined features from both branches to compute a cross-covariance matrix, recalibrating the spatial focus of the LinAngular branch with the channel-wise context of the XCA branch. The final fused output is computed as Equation 3:

$$F_{\text{out}}=F_{\text{LA}}⊗\sigma \left(G\left(F_{\text{XCA}}\right)\right)+F_{\text{XCA}}⊗\sigma \left(G\left(F_{\text{LA}}\right)\right)$$ (3)

where G represents the global context interaction operation, σ denotes the Sigmoid activation function, and ⊗ indicates element-wise multiplication. By integrating this module, the network outputs a recalibrated feature map where lineage-specific characteristics are significantly enhanced through global context modeling.

#### 2.4.2 Gated Convolutional Block (ResGDNet)

Following feature extraction, it is imperative to selectively propagate biologically relevant information while dampening noise, particularly given the low contrast and floating debris typical of label-free brightfield microscopy. To achieve this, we incorporate the Gated CNN Block (Figure 4C). This design draws inspiration from the Gated Linear Units (GLU) originally proposed for language modeling by Dauphin et al. (2017), but we adapt it here for 2D visual feature maps within an inverted bottleneck architecture (Liu et al., 2022).

As depicted in Figure 4C, the input tensor undergoes Layer Normalization to stabilize training dynamics before being projected into a higher-dimensional space via a fully connected (FC1) layer. The flow is then bifurcated into two parallel paths: a content path and a gating path. The gating path acts as a learnable filter, utilizing a projection layer followed by a Gaussian Error Linear Unit (GELU) activation.

The critical operation is the element-wise multiplication of the content path by the gating path. This mechanism enables the network to learn a dynamic feature selection policy: the gate “opens” (values approaching 1) for features strongly correlated with differentiation markers—such as the branching patterns of oligodendrocytes—and “closes” (values approaching 0) for ambiguous background regions. This selective mechanism functionally mimics the noise-suppression capability of the biological visual system. Formally, given an input feature map X , the output $h_{l}$ of the block is defined as Equation 4:

$$h_{l}\;\left(X\right)=\left(X\;*\;W+b\right)⊗\sigma \left(X\;*\;V+c\right),$$ (4)

where W and V are learned kernels for the linear transmission and gating operations, respectively, and ⊗ denotes element-wise multiplication (Dauphin et al., 2017).

In our implementation, to optimize parameter efficiency, we incorporate depthwise convolution within the gating mechanism. The input is partitioned into three components: gate ( g ), information ( i ), and context ( c ). The context component undergoes depthwise convolution, applying a single filter to each input channel (Equation 5):

$$\text{Dept}h\text{wiseConv}{\left(F\right)}_{i,j}=\sum_{k,l}K_{k,l}\cdot F_{i+k,j+l},$$ (5)

where F represents the input feature map and K is the depthwise kernel. By integrating the gating mechanism with depthwise convolution, the model achieves a favorable balance between computational complexity and predictive performance. The gating operation $\sigma \left(X\;*\;V+c\right)$ allows the network to suppress irrelevant background artifacts while retaining high-frequency texture details, effectively functioning as a “soft” attention mechanism. Finally, a residual connection is employed to ensure gradient stability during deep network training.

#### 2.4.3 CG-RecNet: integrated model architecture

The CG-RecNet represents the final, integrated model architecture, built upon the hierarchical feature extraction of the ResNet50 backbone to address the dual challenges of fine-grained morphological classification and noise suppression in label-free imaging. The structure is strategically designed to optimize feature flow by sequentially applying global context modeling and local feature refinement within the core network.

Specifically, the LinAngular-XCA Fusion Module is introduced directly following ResBlock Stage 2. This placement ensures that the feature maps, having acquired reliable hierarchical representations from the initial convolutional stages, are subjected to Global Recalibration. By explicitly modeling long-range spatial and cross-channel dependencies at this mid-level stage, the model gains a comprehensive contextual understanding of the cell morphology, overcoming the local limitations of the early residual layers.

Subsequently, the Gated CNN Block is inserted immediately after the attention module, serving as a critical Local Refinement mechanism. Its function as a learnable filter is to selectively modulate the information flow before features pass into the deeper ResBlock Stage 3 and Stage 4. This sequential placement—hierarchical extraction → global context recalibration → noise-reliable local refinement—ensures that only the most discriminative and noise-free features propagate to the final classification layers. This synergistic integration allows CG-RecNet to achieve reliable lineage prediction superior to its ablation variants.

#### 2.4.4 Comparative models

To validate the specific contributions of our proposed architecture, we established a systematic comparison ranging from the ResNet50 baseline (He et al., 2016), which is limited by fixed receptive fields and standard convolutions, to two progressive variants: ResCMNet, which integrates the LinAngular-XCA Fusion Module to enhance global spatial focus but remains susceptible to noise, and ResGDNet, which employs Gated CNN Blocks to filter background artifacts and refine local textures. Building upon these findings, our proposed CG-RecNet synergistically combines both mechanisms, leveraging dual-branch attention for global context modeling and gated convolutions for noise suppression to address the dual challenges of structural analysis and fine-grained texture recognition, thereby achieving superior classification robustness in label-free stem cell imagery.

To benchmark our proposed method against broader architectural paradigms, we evaluated four state-of-the-art models: DenseNet (Huang et al., 2017), which excels in feature reuse via dense connectivity but suffers from high computational redundancy due to channel concatenation; VGG (Simonyan and Zisserman, 2015), a classic deep network that, despite reliable hierarchical extraction, is limited by excessive parameter volume (approx. 138M) and a lack of global context awareness; MobileNet V2 (Sandler et al., 2018), an efficient architecture renowned for its low computational cost and parameter count, achieved primarily through its inverted residual structure and linear bottlenecks, making it a strong benchmark for deployment efficiency; and Vision Transformer (ViT) (Dosovitskiy et al., 2021), which leverages self-attention for long-range dependencies but struggles with data efficiency and high-frequency local texture capture due to the absence of convolutional inductive biases.

Furthermore, to ensure the high of domain-specific relevance, our comparative suite critically includes the Zhu et al. (2021) Xception-based Model, which first validated the concept of label-free NSC differentiation prediction using the same underlying dataset. The Xception architecture, utilizing depthwise separable convolutions, represents the existing specialized benchmark for efficiency and performance in this domain. The inclusion of this direct competitor is essential to substantiate that CG-RecNet provides a significant architectural advance over the established methodology for accurate and noise-reliable classification of neural stem cell lineages.

### 2.5 Model evaluation metric

The performance of CG-RecNet was evaluated using a comprehensive suite of standard metrics, including Accuracy, Precision, Recall, and the F1-Score, the latter of which provides a balanced assessment of class-imbalanced data. To evaluate discriminative capability across various thresholds, we utilized the Receiver Operating Characteristic (ROC) curve and calculated the macro-average Area Under the Curve (AUC). Statistical significance was assessed using a paired t-test on the 5-fold cross-validation results, with p < 0.05 considered significant. Detailed mathematical formulations for these metrics are provided in the Supplementary Material.

## 3 Results and discussion

### 3.1 Experimental setup and data partitioning

To rigorously evaluate the model’s generalization capability and prevent data leakage, we employed a stratified splitting strategy for dataset partitioning. This approach ensures that the class distribution across all subsets remains balanced while strictly segregating samples to preclude any overlap between the training and evaluation sets. Specifically, the dataset was partitioned to allocate 70% for model training, 15% for validation, and 15% as an independent test set. The validation set was utilized to monitor model convergence, facilitate hyperparameter tuning, and implement early stopping mechanisms. The resulting distribution structure comprises independent training, validation, and testing sets. It is crucial to clarify that while the final application of CG-RecNet is “label-free” (using only brightfield images for inference), the ground truth labels for training the Cross-Entropy Loss function were established using gold-standard immunofluorescent staining during dataset preparation. To ensure the reproducibility of our results, Table 2 summarizes the detailed implementation parameters and training configurations. The proposed model was implemented using the PyTorch framework (version 1.12.0) and trained on a workstation equipped with a 13th Gen Intel Core i7-13620H CPU and an NVIDIA GeForce RTX 4050 Laptop GPU (6 GB VRAM). We employed the Adam optimizer for parameter updates, selected for its efficacy in handling sparse gradients and adaptive learning rates. The training process was conducted over 100 epochs with a batch size of 8. Cross-Entropy was utilized as the loss function. To mitigate overfitting, we adopted the StepLR learning rate decay strategy, configured to decay the learning rate by a factor of 0.1 every 7 epochs.

**TABLE 2:** Model hyperparameters and training Configuration.

Parameter	Configuration/Value
Input resolution	224×224×3 pixels
Model architecture	ResNet50 + LinAngular-XCA fusion + gated CNN
Optimizer	Adam ( $\beta_{1}=0.9,\beta_{2}=0.999$ ; $\epsilon =10^{-8}$ )
Initial learning rate	$1\times 10^{-4}$
Learning rate schedule	ReduceLROnPlateau (factor 0.1, patience 5)
Batch size	8
Total epochs	100
Loss function	Cross-entropy loss
Activation functions	ReLU (intermediate), softmax (final classification)
Hardware	Intel core i7-13620H/RTX 4050 (6 GB)

To ensure the reproducibility of our results, all experiments were conducted using multiple fixed random seeds (seeds = 42, 123, and 999) to verify that the performance gains of CG-RecNet are not artifacts of hyperparameter selection. The reported p-values (p < 0.001) confirm that the architectural improvements are statistically significant regardless of initial weight initialization.

### 3.2 Results of the model

#### 3.2.1 Results of the ablation study

To systematically evaluate the contribution of each proposed module—specifically the GatedCNN module and the LinAngular-XCA fusion mechanism—we conducted an ablation study. We compared the performance of the Baseline model (ResNet50), the model with only the GatedCNN module (ResGDNet), the model with only the LinAngular-XCA module (ResCMNet), and the proposed full framework (CG-RecNet). The quantitative results, presented as mean ± standard deviation across 5-fold cross-validation, are summarized in Table 3.

**TABLE 3:** Result of the test.

Model	Accuracy	Precision	Recall	F1	Total parameters (M)	GFLOPs
CG-RecNet	96.40%±0.07%	96.38%±0.19%	96.40%±0.06%	96.39%±0.05%	**28.02**	**8.40**
ResCMNet	96.32%±0.11%	96.29%±0.15%	96.32%±0.09%	96.30%±0.07%	27.69	8.19
ResGDNet	95.63%±0.08%	95.58%±0.25%	95.63%±0.05%	95.60%±0.06%	25.89	7.73
Baseline	94.58%±0.08%	94.50%±0.19%	94.58%±0.12%	94.47%±0.07%	25.51	6.13

Bold values indicate the best performance metrics among the compared models.

**FIGURE 5:**
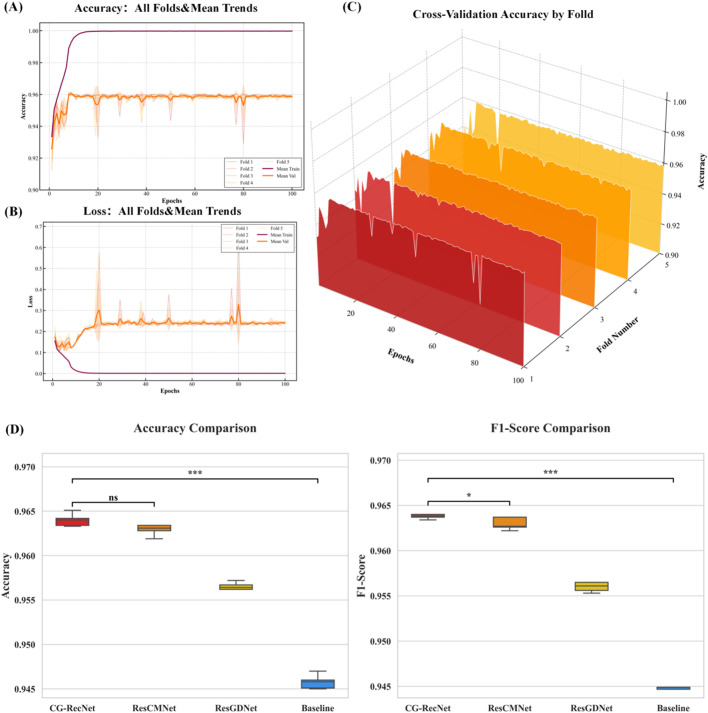
Five-Fold Cross-Validation Performance Metrics and Training Dynamics for the CG-RecNet Model. **(A)** Accuracy trajectory across all five folds. **(B)** Loss convergence curves. **(C)** Detailed training dynamics for a representative fold (Fold 5). **(D)** Statistical distribution of performance metrics.

The visualization in Figure 5 provides a assessment of the CG-RecNet’s stability and learning process across five distinct data partitions. (A) Illustrates the trajectory of the accuracy metric during the 5-fold cross-validation process. The bold solid lines denote the mean training and validation accuracies across the five folds, while the shaded regions represent the standard deviation. The thin lines in the background display the specific performance of each individual fold (Fold 1–5), effectively reflecting the model’s stability across distinct data partitions. (B) Presents the corresponding loss convergence curves. The consistent decline in both training and validation losses over the epochs indicates strong convergence capabilities, with no significant evidence of overfitting. (C) Provides a detailed training overview of Fold 5. As a representative fold, this plot further indicates the close alignment between training and validation metrics.

To rigorously validate the statistical reliability of these results, (D) presents the box plots of Accuracy and F1-Score distributions. Unlike the trajectories, this panel explicitly highlights the variance and median performance, with the Student’s t-test results ( p < 0.001 ) confirming a statistically significant improvement of CG-RecNet over the Baseline. Collectively, these indicators substantiate the model’s reliable performance and generalization capability on the stem cell dataset.

**FIGURE 6:**
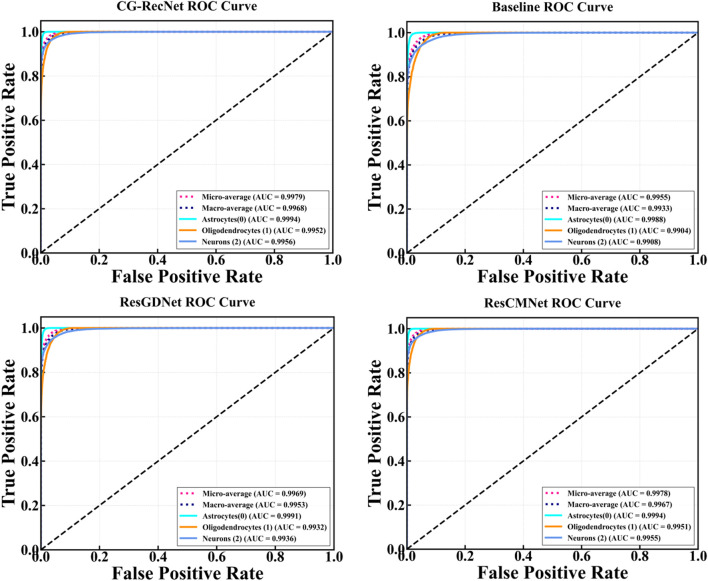
Comparative Receiver Operating Characteristic (ROC) curves for Various Models.

Figure 6 displays the ROC curves comparing the diagnostic performance of CG-RecNet, Baseline, ResGDNet, and ResCMNet architectures. The CG-RecNet curves are notably positioned closest to the top-left corner across all classes, indicating the model’s superior capability to distinguish between different categories and achieving the highest diagnostic accuracy (AUC) and model stability.

**FIGURE 7:**
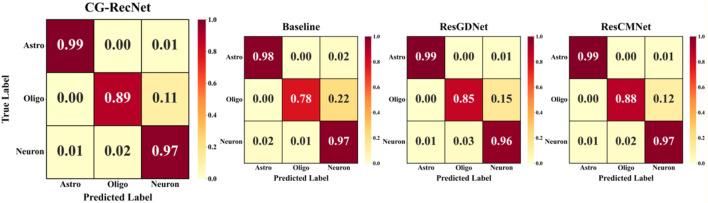
Normalized confusion matrices.

Figure 7 displays the normalized confusion matrices for the CG-RecNet, Baseline, ResGDNet, and ResCMNet architectures. The x-axis represents the predicted labels, while the y-axis represents the true labels. Class labels are defined as: 0 = Astro, 1 = Oligo, and 2 = Neuron. The matrix for CG-RecNet reveals a dense concentration of samples along the main diagonal, achieving 99% accuracy for Astrocytes, 89% for the minority Oligodendrocytes, and 97% for Neurons. This visualization demonstrates the model’s high predictive precision and low inter-class confusion compared to the Baseline and ablation variants on the internal dataset.

The diagnostic efficacy of CG-RecNet was further evaluated in relation to computational complexity to assess the trade-off between performance and resource cost. As detailed in Table 3, the integration of novel components led to progressive performance gains. The introduction of the attention mechanism (ResCMNet) resulted in a notable accuracy increase from the Baseline’s 94.58% ± 0.08% to 96.32% ± 0.11%. Although this addition increased computational cost (from 6.13 GFLOPs to 8.19 GFLOPs) due to global context modeling, the subsequent integration of the Gated CNN Block in the final CG-RecNet framework achieved the peak accuracy of 96.40% ± 0.07%. Statistical analysis confirmed that the improvement over the Baseline was statistically significant (p < 0.001).

Regarding model complexity, the final integrated architecture (CG-RecNet) utilizes 28.02 M parameters and 8.40 GFLOPs. This 1.82% absolute improvement in accuracy is accompanied by a ∼9.8% increase in model parameters (from 25.51 M to 28.02 M) and a ∼37% increase in GFLOPs (from 6.13 to 8.40). While CG-RecNet is more efficient than older, high-parameter architectures like VGG-16, the baseline ResNet50 may offer a superior balance between predictive capability and computational resource consumption—providing a better ‘bang for the buck'—for users with limited hardware resources.

#### 3.2.2 Results of the comparison experiments

To strictly evaluate the performance of the proposed method, we conducted comparative experiments against several mainstream deep learning models, including DenseNet (Huang et al., 2017), VGG (Simonyan and Zisserman, 2015), Vision Transformer (VIT) (Dosovitskiy et al., 2021) and MobileNet V2 (Sandler et al., 2018). The quantitative results are presented in Table 3, focusing on four key evaluation metrics: Accuracy, Precision, Recall, and F1-score.

As presented in Table 4, the proposed CG-RecNet achieved superior performance across all evaluation metrics, demonstrating its robustness in the classification task. Specifically, CG-RecNet attained the highest Accuracy of 96.40% ± 0.07%, Precision of 96.38% ± 0.19%, Recall of 96.40% ± 0.06%, and F1-score of 96.39% ± 0.05%. Notably, our model outperformed the second-best architecture, MobileNet V2, which recorded an accuracy of 96.07% ± 0.12% and an F1-score of 96.04% ± 0.09%. DenseNet and VGG followed closely with accuracies of 96.04% ± 0.14% and 95.82% ± 0.18%, respectively, whereas the ViT model yielded a comparatively lower accuracy of 94.27% ± 0.25%. These quantitative results—highlighting both superior mean performance and lower variance—substantiate the efficacy of the proposed architectural enhancements, confirming that CG-RecNet offers a highly reliable solution relative to established baselines.

**TABLE 4:** Result of the Comparison experiments.

Model	Accuracy	Precision	Recall	F1
CG-RecNet	96.40%±0.07%	96.38%±0.19%	96.40%±0.06%	96.39%±0.05%
MobileNet V2	96.07%±0.12%	96.03%±0.15%	96.07%±0.10%	96.04%±0.09%
DenseNet	96.04%±0.14%	96.01%±0.18%	96.04%±0.11%	96.02%±0.10%
VGG	95.82%±0.18%	95.77%±0.22%	95.82%±0.15%	95.78%±0.14%
VIT	94.27%±0.25%	94.17%±0.30%	94.27%±0.22%	94.18%±0.21%

Bold values indicate the best performance metrics among the compared models.

While the overall accuracy improvement is incremental compared to MobileNet V2 (96.07% ± 0.12%), a more distinct advantage is observed in lineage-specific performance. To provide a more comprehensive assessment, Table 5 compares the per-class efficacy of CG-RecNet against both the baseline and all other SOTA architectures. As demonstrated, the superior performance of CG-RecNet is underscored by its ability to consistently outperform all competitive models across every neural lineage.

**TABLE 5:** Per-class diagnostic performance and lineage classification efficacy.

Class id	Cell type	Support	ViT	VGG	DenseNet	MobileNet V2	Baseline	CG-RecNet	CG-RecNet improvement
0	Astrocytes	8,320	95.80%	97.82%	98.13%	98.15%	97.40%	98.34%	**+0.94%**
1	Oligodendrocytes	4,153	81.15%	85.12%	86.45%	87.20%	83.81%	89.75%	**+5.94%**
2	Neurons	18,661	94.60%	96.10%	96.33%	96.55%	95.53%	96.99%	**+1.46%**

Bold values indicate the best performance metrics among the compared models.

Notably, our model achieves a substantial +5.94% F1-Score improvement in the challenging minority Oligodendrocyte lineage (F1 = 89.75%) compared to the baseline (83.81%), and maintains a clear margin over other advanced models such as MobileNet V2 (87.20%) and DenseNet (86.45%). This gain is particularly significant as oligodendrocytes are crucial for remyelination and the treatment of neurological diseases like Multiple Sclerosis, a minority class that is morphologically difficult to identify. Alongside this, the model showed strong performance in Astrocytes (F1 = 98.34%) and Neurons (F1 = 96.99%), exceeding the diagnostic precision of all benchmarked architectures. This differential success confirms that the synergistic integration of the attention module and the Gated CNN effectively extracts highly discriminative morphological features, ensuring high precision across all three biologically critical lineages while effectively overcoming the classification bias inherent in generic models.

In addition to benchmarking against general SOTA architectures (Table 4), we critically evaluated CG-RecNet against the established domain-specific method: the Xception-based model by Zhu et al. (2021), which first validated the potential for label-free NSC prediction using the same core dataset. The original Xception-based model achieved an accuracy of 92.3% on comparable brightfield test data. CG-RecNet’s overall accuracy of 96.40% represents a substantial performance margin over this foundational domain-specific benchmark. This significant improvement stems from CG-RecNet’s architectural advancements, which directly address the core limitations of standard CNNs like Xception. While the Xception-based model relies on depthwise separable convolutions to focus on local features and lacks dedicated noise control, CG-RecNet introduces two complementary mechanisms: the LinAngular-XCA Fusion Module to capture long-range morphological dependencies and global context, and the Gated CNN Block to act as a dynamic, learnable filter for noise suppression and local texture refinement. This synergistic, hybrid design proves essential for extracting the fine-grained morphological features necessary for high-precision classification in complex brightfield environments, confirming that CG-RecNet provides a significant architectural and predictive advance over the established methodology.

### 3.3 Qualitative interpretability analysis via grad-CAM

To explore the visual focus of CG-RecNet during the classification of neural stem cell differentiation, we conducted a qualitative examination using Gradient-weighted Class Activation Mapping (Grad-CAM). This method provides a visual approximation of the image regions that contribute most significantly to the model’s categorical predictions.

**FIGURE 8:**
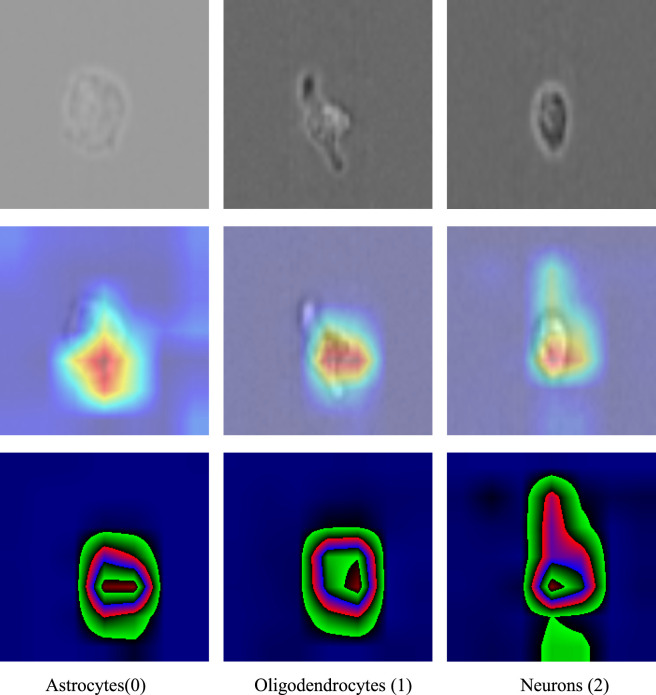
Visualization of class activation mappings (Grad-CAM). Representative visualization results for Astrocytes (0), Oligodendrocytes (1), and Neurons (2). The heatmaps qualitatively illustrate the focus areas of the CG-RecNet model. Scale bar = 10 um.

As shown in Figure 8, the heatmaps generated by CG-RecNet indicate that the model’s attention is primarily concentrated on regions characterized by high pixel intensity variations and specific morphological textures within the brightfield imagery. For instance, in neuronal samples, the activation areas often align with the elongated structures and high-contrast boundaries of the cells.

It is important to emphasize that this analysis represents a qualitative method to visualize feature importance rather than a definitive biological validation of cellular structures. While the heatmaps suggest that the model prioritizes relevant morphological regions, these observations are based on a limited set of representative samples. The alignment between the model’s attention and visual cellular features serves as a diagnostic aid for inspecting the decision-making rationale, demonstrating that the network captures salient phenotypic patterns to differentiate between Astrocyte, Oligodendrocyte, and Neuron lineages.

The figure presents representative visualization results for three distinct stem cell categories, sequentially labeled as (0), (1), and (2). The top row displays the original microscopic images, while the lower rows illustrate the corresponding attention heatmaps. These heatmaps visually delineate the focus areas of the CG-RecNet model, where high-activation regions (highlighted in red and yellow) visually align with cover the morphological structures of the stem cells. This indicates that the model effectively prioritizes relevant biological features over background noise during classification, thereby revealing the model’s decision-making rationale and validating its interpretability in cellular analysis.

As discussed in the limitations, while Grad-CAM provides visual evidence of the model’s focus, these findings remain qualitative. The heatmaps identify salient pixel intensity patterns rather than providing a quantitative biological correlation, a distinction that is crucial for interpreting the model’s decision-making rationale.

## 4 Discussion

### 4.1 Overview and rationale for the CG-RecNet framework

This study validates CG-RecNet, a specialized hybrid deep learning framework engineered for the accurate, non-invasive, and label-free multi-class classification of Neural Stem Cell (NSC) differentiation lineages. Precise monitoring of neurogenesis is paramount for translational regenerative medicine, yet the reliance on immunofluorescent staining remains a significant bottleneck, introducing cellular toxicity and precluding longitudinal analysis. CG-RecNet addresses this methodological challenge by extracting fine-grained, lineage-specific morphological features directly from ubiquitous brightfield microscopy images.

The framework’s performance is rooted in its deliberate architecture, designed to overcome two primary technical difficulties inherent in label-free cellular imaging: the low contrast and high noise of the background, and the visual ambiguity in distinguishing closely related cell types. By integrating the hierarchical feature extraction of a ResNet50 backbone with two dedicated modules—the LinAngular Cross-Channel Attention (LinAngular-XCA) Fusion Module for global context modeling and the Gated Convolutional Neural Network (GatedCNN) Block for local feature refinement—CG-RecNet establishes a new benchmark for accuracy in this domain.

### 4.2 Comparative performance analysis and architectural superiority

The empirical results demonstrate the enhanced predictive capacity of CG-RecNet on the internal dataset, achieving an overall accuracy of 96.40%. As illustrated in Table 3, the 1.82% accuracy gain over the ResNet50 baseline requires a ∼9.8% increase in parameter count. While VGG-16 is significantly larger, it belongs to an older architectural paradigm and may not serve as a direct benchmark for modern deployment. For applications where resource constraints are paramount, the baseline model may be preferable as it provides the ‘biggest bang for the buck,’ yielding only a 1.8% lower accuracy for a more compact model size. The additional complexity of CG-RecNet is justified primarily in scenarios where high-precision identification of minority lineages, such as oligodendrocytes, is the critical requirement. This efficiency is critical for laboratory deployment, offering an optimal trade-off between SOTA-level precision and the computational accessibility required for standard workstations.

Specifically, CG-RecNet surpassed established CNNs like VGG and MobileNet V2, and showed competitive advantages over modern models such as Vision Transformer (ViT) and DenseNet. For instance, the ViT model yielded a comparatively lower accuracy of 94.27% ± 0.25%. This deficit can be attributed to the ViT’s inherent challenge in capturing the high-frequency local texture and fine cellular process features crucial for cell typing, owing to its lack of explicit convolutional inductive bias. In contrast, CG-RecNet maintains the benefits of convolution while enhancing global perception.

Our model’s marginal yet critical improvement over the optimized DenseNet (96.04% ± 0.14% accuracy) is attributable to the strategic functional separation within our hybrid design. DenseNet may not optimally filter out the non-informative background artifacts common in brightfield images. CG-RecNet’s integrated Gated CNN Block acts as a selective noise filter, refining local feature maps to retain biologically relevant information, thereby increasing the signal-to-noise ratio of the input features.

Furthermore, CG-RecNet achieved a +4.1% performance margin compared to the domain-specific benchmark using an Xception-based architecture (Zhu et al., 2021). This suggests that architectures relying primarily on local operations struggle to integrate the long-range morphological dependencies (e.g., the extent of neurite outgrowth) essential for high-fidelity classification. The introduction of the LinAngular-XCA Fusion Module specifically resolves this by efficiently capturing non-local, long-range cellular morphology features.

### 4.3 Robustness against class imbalance and biological significance

A key finding of this study is the high classification efficacy of CG-RecNet on imbalanced NSC datasets, particularly regarding minority lineages. Datasets derived from biological differentiation often exhibit natural skewness, particularly regarding terminally differentiated cell types. In our case, the oligodendrocyte lineage constitutes a critical but minority class. Conventional deep learning models frequently suffer from classification bias towards the majority classes.

Notably, CG-RecNet achieved a +5.94% F1-Score improvement for the critical Oligodendrocyte lineage (F1-Score of 89.75%) compared to the Baseline model (83.81%). This result represents the primary contribution of our work. Biologically, oligodendrocytes are the “minority class” and are notoriously difficult to distinguish from background noise due to their subtle morphology compared to the abundant neurons. However, in the context of regenerative medicine for demyelinating diseases (e.g., Multiple Sclerosis), the ability to accurately identify these rare therapeutic cells is the critical bottleneck, far more valuable than marginal gains in detecting already abundant neurons. Standard models often sacrifice the accuracy of these minority classes to achieve a high “average” score. By selectively suppressing background artifacts through the Gated CNN, CG-RecNet effectively “closes the gap” on this hardest class, offering a diagnostic reliability that generic models may not consistently provide.

### 4.4 Interpretability and translational potential

Addressing the “black box” challenge in biological applications, CG-RecNet incorporates *post hoc* interpretability through Grad-CAM visualization. By generating activation maps, we provided a rationale for the model’s decision-making process. As illustrated in Figure 8, the model consistently focuses on biologically relevant morphological hallmarks—such as the length of neurite extensions in neurons, or the textured appearance of the soma in glial cells—that align closely with established histological criteria used by human experts.

This interpretability transforms CG-RecNet from a purely predictive tool into a transparent diagnostic aid. It facilitates human-AI collaboration for validating observed biological phenomena and accelerating phenotypic drug screening (Bekhite and Schulze, 2021). While molecular assays remain the gold standard, CG-RecNet serves as a scalable, cost-effective surrogate marker for automated, routine monitoring in culture.

### 4.5 Limitations and future directions

Despite the encouraging results, this study acknowledges methodological limitations.

First, the model was trained and validated on a single publicly available dataset derived from rat embryonic NSCs. While we employed a stratified cross-validation strategy to mitigate overfitting, the lack of an external, independent dataset (out-of-context validation) limits the assessment of the model’s generalization capability. Although our architectural principles of global-local fusion are designed to handle high noise, cellular heterogeneity among human iPSC lines (Kilpinen et al., 2017) and variations in imaging protocols across different laboratories remain significant challenges for biological translation. Future work will focus on expanding the training cohort to include multi-institutional data to rigorously verify cross-platform generalization.

Second, the data utilized in this study were acquired via Imaging Flow Cytometry (IFC), which provides high-throughput, pre-segmented single-cell images. Consequently, the current framework focuses on phenotypic classification and does not address the challenges of cell segmentation or regional identification required for standard adherent cell microscopy. Adapting CG-RecNet for *in situ* microscopy applications would necessitate the integration of an additional upstream segmentation module.

Third, the current study utilizes fixed time-point brightfield images and does not yet incorporate the continuous temporal dynamics inherent to live-cell differentiation. Cell fate is a dynamic trajectory, and leveraging temporal information can enhance the prediction of differentiation outcomes at earlier stages. Therefore, a crucial direction involves integrating continuous video data into a spatiotemporal deep learning framework. This will allow CG-RecNet to analyze differentiation kinetics, contributing to the development of a holistic intelligent monitoring system for practical regenerative medicine research.

Fourth, while the Grad-CAM visualizations provide valuable insights into the model’s decision-making process, this interpretability analysis remains primarily qualitative. Due to the high-throughput nature of the imaging flow cytometry dataset and the inherent complexity of defining pixel-level spatial ground truths for all morphological artifacts, a large-scale quantitative evaluation of heatmap accuracy was not performed in this study. Future research will aim to develop standardized quantitative metrics to further validate the precision of these attention-based focus areas in label-free biological contexts.

## 5 Conclusion

This study successfully introduces and validates CG-RecNet, a reliable, hybrid deep learning architecture specifically designed for the accurate, label-free multi-class classification of neural stem cell differentiation lineages. By synergistically combining the hierarchical feature extraction capabilities of ResNet50 with the enhanced global context modeling of the LinAngular-XCA Fusion Module and the noise-suppression mechanisms of Gated CNN Blocks, the proposed model effectively addresses the inherent challenges of distinguishing subtle morphological phenotypes in complex brightfield microscopy images. Our extensive empirical evaluation on a diverse dataset confirms the reliable diagnostic performance of CG-RecNet, which achieved an overall accuracy of 96.40% and an average AUC of 0.9979. This performance margin surpasses that of several established baseline architectures, including advanced models like ViT and DenseNet. Furthermore, the strategic integration of Grad-CAM visualizations successfully mitigates the “black-box” nature of the deep learning model, providing transparent visual evidence of the specific cell morphological features driving the prediction. This visualization provides qualitative evidence of the morphological features driving the classification, suggesting the potential for enhanced diagnostic transparency in automated quality control systems. Future research will focus on extending the model’s applicability by validating CG-RecNet on large-scale, multi-institutional human iPSC-derived datasets to ensure reliable generalization across diverse cell lines and imaging platforms. Additionally, we aim to explore the integration of temporal dynamic data, extending the model to analyze continuous video streams of cell growth. This next step is essential for enabling the prediction of differentiation trajectories at earlier stages, thereby contributing to the development of a holistic intelligent monitoring system crucial for the advancement of regenerative medicine manufacturing.

## Data Availability

The original contributions presented in the study are included in the article/Supplementary Material, further inquiries can be directed to the corresponding author.

## References

[B1] Bekhite M. M. Schulze P. C. (2021). Human induced pluripotent stem cell as a disease modeling and drug development Platform-A cardiac perspective. Cells 10 (12), 3483. 10.3390/cells10123483 34943991 PMC8699880

[B2] Bradbury E. J. Burnside E. R. (2019). Moving beyond the glial scar for spinal cord repair. Nat. Commun. 10 (1), 1–13. 10.1038/s41467-019-11707-7 31462640 PMC6713740

[B3] Christiansen E. M. Yang S. J. Ando D. M. Javaherian A. Skibinski G. Lipnick S. (2018). *In silico* labeling: predicting fluorescent labels in unlabeled images. Cell 173 (3), 792–803. 10.1016/j.cell.2018.03.040 29656897 PMC6309178

[B4] Cieri M. B. Ramos A. J. (2025). Astrocytes, reactive astrogliosis, and glial scar formation in traumatic brain injury. Neural Regen. Res. 20 (4), 973–989. 10.4103/nrr.nrr-d-23-02091 38989932 PMC11438322

[B5] Dauphin Y. N. Fan A. Auli M. Grangier D. (2017). “Language modeling with gated convolutional networks,” in International conference on machine learning (Brookline, MA: PMLR), 933–941.

[B6] De Gioia R. Biella F. Citterio G. Rizzo F. Abati E. Nizzardo M. (2020). Neural stem cell transplantation for neurodegenerative diseases. Int. J. Mol. Sci. 21 (9), 3103. 10.3390/ijms21093103 32354178 PMC7247151

[B7] Dimou L. Götz M. (2014). Glial cells as progenitors and stem cells: new roles in the healthy and diseased brain. Physiol. Rev. 94 (3), 709–737. 10.1152/physrev.00036.2013 24987003

[B8] Dosovitskiy A. Beyer L. Kolesnikov A. (2021). “An image is worth 16x16 words: transformers for image recognition at scale,” in International conference on learning representations (ICLR).

[B9] El-Nouby A. Touvron H. Caron M. (2021). XCiT: cross-covariance image transformers. Adv. Neural Inf. Process. Syst. (NeurIPS). 10.48550/arXiv.2106.09681

[B10] Esteva A. Kuprel B. Novoa R. A. Ko J. Swetter S. M. Blau H. M. (2017). Dermatologist-level classification of skin cancer with deep neural networks. Nature 542 (7639), 115–118. 10.1038/nature21056 28117445 PMC8382232

[B11] Fawcett J. W. Asher R. A. (1999). The glial scar and central nervous system repair. Brain Res. Bull. 49 (6), 377–391. 10.1016/s0361-9230(99)00072-6 10483914

[B12] Feigin V. L. Nichols E. Alam T. (2019). Global, regional, and national burden of neurological disorders, 1990–2016: a systematic analysis for the global burden of disease study 2016. Lancet Neurology 18 (5), 459–480. 10.1016/S1474-4422(18)30499-X 30879893 PMC6459001

[B13] Franklin R. J. Bodini B. Goldman S. A. (2024). Remyelination in the central nervous system. Cold Spring Harb. Perspect. Biol. 16 (3), a041371. 10.1101/cshperspect.a041371 38316552 PMC10910446

[B14] Fujitani M. Huddin N. S. Kawai S. Kanie K. Kiyota Y. Shimizu K. (2017). Morphology-based non-invasive quantitative prediction of the differentiation status of neural stem cells. J. Bioscience Bioengineering 124 (3), 351–358. 10.1016/j.jbiosc.2017.04.006 28465021

[B15] Gao L. Peng Y. Xu W. He P. Li T. Lu X. (2020). Progress in stem cell therapy for spinal cord injury. Stem Cells Int. 2020, 2853650. 10.1155/2020/2853650 33204276 PMC7661146

[B16] Hauser S. L. Oksenberg J. R. (2006). The neurobiology of multiple sclerosis: genes, inflammation, and neurodegeneration. Neuron 52 (1), 61–76. 10.1016/j.neuron.2006.09.011 17015227

[B17] He K. Zhang X. Ren S. Sun J. (2016). Deep residual learning for image recognition. Proc. IEEE Conf. Comput. Vis. Pattern Recognit. (CVPR), 770–778. 10.1109/CVPR.2016.90

[B18] Huang G. Liu Z. Van Der Maaten L. Weinberger K. Q. (2017). “Densely connected convolutional networks,” in Proceedings of the IEEE Conference on Computer Vision and Pattern Recognition (CVPR), Honolulu, HI, USA, 21-26 July 2017 (IEEE), 4700–4708.

[B19] Jia H. Zhang J. Ma K. Qiao X. Ren L. Shi X. (2024). Application of convolutional neural networks in medical images: a bibliometric analysis. Quantitative Imaging Med. Surg. 14 (5), 3501–3518. 10.21037/qims-23-1600 38720828 PMC11074758

[B20] Jucker M. Walker L. C. (2018). Propagation and spread of pathogenic protein assemblies in neurodegenerative diseases. Nat. Neurosci. 21 (10), 1341–1349. 10.1038/s41593-018-0238-6 30258241 PMC6375686

[B21] Kalia L. V. Lang A. E. (2015). Parkinson's disease. Lancet 386 (9996), 896–912. 10.1016/S0140-6736(14)61393-3 25904081

[B22] Kilpinen H. Goncalves A. Leha A. Afzal V. Alasoo K. Ashford S. (2017). Common genetic variation drives molecular heterogeneity in human iPSCs. Nature 546 (7658), 370–375. 10.1038/nature22403 28489815 PMC5524171

[B23] Krikid F. Rositi H. Vacavant A. (2024). State-of-the-Art deep learning methods for microscopic image segmentation: applications to cells, nuclei, and tissues. J. Imaging 10 (12), 311. 10.3390/jimaging10120311 39728208 PMC11679639

[B24] Linnerbauer M. Wheeler M. A. Quintana F. J. (2020). Astrocyte crosstalk in CNS inflammation. Neuron 108 (4), 608–622. 10.1016/j.neuron.2020.08.012 32898475 PMC7704785

[B25] Litjens G. Kooi T. Bejnordi B. E. Setio A. A. A. Ciompi F. Ghafoorian M. (2017). A survey on deep learning in medical image analysis. Med. Image Anal. 42, 60–88. 10.1016/j.media.2017.07.005 28778026

[B27] Li C. Luo Y. Li S. (2024). The roles of neural stem cells in myelin regeneration and repair therapy after spinal cord injury. Stem Cell Res. Ther. 15, 204. 10.1186/s13287-024-03825-x 38978125 PMC11232222

[B26] Liu Z. Mao H. Wu C. (2022). “A ConvNet for the 2020s,” in Proceedings of the IEEE/CVF conference on computer vision and pattern recognition (CVPR), 11976–11986.

[B28] Long J. M. Holtzman D. M. (2019). Alzheimer disease: an update on pathobiology and treatment strategies. Cell 179 (2), 312–339. 10.1016/j.cell.2019.09.001 31564456 PMC6778042

[B29] Mao J. He H. (2024). Deep learning in fluorescence imaging and analysis. J. Intelligent Med. 1 (1), 42–62. 10.1002/jim4.17

[B30] Martino G. Pluchino S. Bonfanti L. Schwartz M. (2011). Brain regeneration in physiology and pathology: the immune signature driving therapeutic plasticity of neural stem cells. Physiol. Rev. 91 (4), 1281–1304. 10.1152/physrev.00032.2010 22013212 PMC3552310

[B31] Moen E. Bannon D. Kudo T. Graf W. Covert M. Van Valen D. (2019). Deep learning for cellular image analysis. Nat. Methods 16 (12), 1233–1246. 10.1038/s41592-019-0403-1 31133758 PMC8759575

[B32] Sandler M. Howard A. Zhu M. (2018). “MobileNetV2: inverted residuals and linear bottlenecks,” in Proceedings of the IEEE Conference on Computer Vision and Pattern Recognition (CVPR), Salt Lake City, UT, USA, 18-23 June 2018 (IEEE), 4510–4520.

[B33] Shorten C. Khoshgoftaar T. M. (2019). A survey on image data augmentation for deep learning. J. Big Data 6 (1), 1–48. 10.1186/s40537-019-0197-0 PMC8287113 34306963

[B34] Simonyan K. Zisserman A. (2015). “Very deep convolutional networks for large-scale image recognition,” in International conference on learning representations (ICLR).

[B35] Siracusa R. Fusco R. Cuzzocrea S. (2019). Astrocytes: role and functions in brain pathologies. Front. Pharmacol. 10, 1114. 10.3389/fphar.2019.01114 31611796 PMC6777416

[B36] Sofroniew M. V. (2009). Molecular dissection of reactive astrogliosis and glial scar formation. Trends Neurosci. 32 (12), 638–647. 10.1016/j.tins.2009.08.002 19782411 PMC2787735

[B37] Varma S. Simon R. (2006). Bias in error estimation when using cross-validation for model selection. BMC Bioinformatics 7 (1), 91. 10.1186/1471-2105-7-91 16504092 PMC1397873

[B38] Vaswani A. Shazeer N. Parmar N. (2017). Attention is all you need. Adv. Neural Inf. Process. Syst. 30. 10.48550/arXiv.1706.03762

[B39] Warnock A. Toomey L. M. Wright A. J. Fisher K. Won Y. Anyaegbu C. (2020). Damage mechanisms to oligodendrocytes and white matter in central nervous system injury: the Australian context. J. Neurotrauma 37 (5), 739–769. 10.1089/neu.2019.6890 32027208

[B40] Woo S. Park J. Lee J. Y. Kweon I. S. (2018). “CBAM: convolutional block attention module,” in Proceedings of the European conference on computer vision (Cham, Switzerland: Springer), 3–19.

[B41] Xue C. R. Wang K. Zhang M. Z. Wang Z. Song Y. Y. Yu H. J. (2022). Tracking neural stem cells *in vivo:* achievements and limitations. Stem Cell Rev. Rep. 18 (5), 1774–1788. 10.1007/s12015-022-10333-z 35122628

[B42] Yang W. Lv M. Yu Z. Deng J. (2025). A wavelet-guided transformer approach for autofocus in brightfield biological microscopy. Sci. Rep. 15 (1), 25521. 10.1038/s41598-025-11037-3 40665153 PMC12264130

[B43] Zhou S. K. Greenspan H. Davatzikos C. Duncan J. S. Van Ginneken B. Madabhushi A. (2021). A review of deep learning in medical imaging: imaging traits, technology trends, case studies with progress highlights, and future promises. Proc. IEEE 109 (5), 820–838. 10.1109/jproc.2021.3054390 37786449 PMC10544772

[B44] Zhou H. Luo F. Zhuang H. Weng Z. Gong X. Lin Z. (2023a). Attention multi-hop graph and multi-scale convolutional fusion network for hyperspectral image classification. IEEE Trans. Geoscience Remote Sens. 61, 1–13. 10.1109/tgrs.2023.3265879

[B45] Zhou H. Luo F. Zhuang H. Weng Z. Gong X. Lin Z. (2023b). Attention multihop graph and multiscale convolutional fusion network for hyperspectral image classification. IEEE Trans. Geoscience Remote Sens. 61, 1–14. 10.1109/TGRS.2023.3265879

[B46] Zhu Y. Huang R. Wu Z. Song S. Cheng L. Zhu R. (2021). Deep learning-based predictive identification of neural stem cell differentiation. Nat. Commun. 12 (1), 2614. 10.1038/s41467-021-22758-0 33972525 PMC8110743

