# Supplementary material for: CG-RecNet: a gated and attention-fused deep learning framework for label-free classification of neural stem cell differentiation via imaging flow cytometry
Source: Front Cell Dev Biol. 2026 Feb 16;14:1767574. doi: 10.3389/fcell.2026.1767574 (PMC12950795; doi:10.3389/fcell.2026.1767574)
Supplement: Supplementary file 1 [file DataSheet1.docx]

# Supplementary Material

To evaluate the efficacy of machine learning models, particularly within the domain of cellular image analysis, the employment of rigorous evaluation metrics is imperative. This study utilizes a comprehensive suite of metrics derived from the fundamental elements of the confusion matrix: True Positives (TP), False Negatives (FN), True Negatives (TN), and False Positives (FP). Accuracy serves as a fundamental metric, quantifying the proportion of correctly predicted observations relative to the total number of observations. Precision denotes the ratio of correctly predicted positive observations to the total predicted positives, whereas Recall (or Sensitivity) measures the proportion of correctly predicted positive observations against all observations in the actual class. The F1-score represents the harmonic mean of Precision and Recall, providing a balanced evaluation metric particularly valuable in scenarios involving class imbalance. The mathematical formulations for these metrics are defined as follows:

$$\begin{aligned} Accuracy=\frac{TP+TN}{TP+TN+FP+FN}\#\left( \text{6} \right) \end{aligned}$$

$$\begin{aligned} Precision=\frac{\mathrm{TP}}{TP+FP}\#\left( \text{7} \right) \end{aligned}$$

$$\begin{aligned} Recall=\frac{\mathrm{TP}}{TP+FN}\#\left( \text{8} \right) \end{aligned}$$

$$\begin{aligned} F1\text{-}Score=2\times\frac{Precision\times Recall}{Precision+Recall}\#\left( \text{9} \right) \end{aligned}$$

Furthermore, to evaluate the model's ability to discriminate between classes at various threshold settings, we utilize the Receiver Operating Characteristic (ROC) curve. The ROC curve plots the True Positive Rate (TPR) against the False Positive Rate (FPR), which are defined as:

$$\begin{aligned} TPR=\frac{\mathrm{TP}}{TP+FN}\#\left( \text{10} \right) \end{aligned}$$

$$\begin{aligned} FPR=\frac{\mathrm{FP}}{FP+TN}\#\left( \text{11} \right) \end{aligned}$$

The Area Under the Curve (AUC) provides a singular scalar value summarizing the model's performance across all classification thresholds. Mathematically, the AUC is calculated as the integral of the ROC curve, where $TPR(t)$ and $FPR(t)$ are functions of the threshold parameter $t$:

$$\begin{aligned} AUC=\int_{0}^{1} \mathrm{TPR}\left( \mathrm{FP}R^{-1}\left( x \right) \right)dx\#\left( \text{12} \right) \end{aligned}$$

An AUC value of 1.0 indicates perfect discrimination, while 0.5 suggests no discriminative ability. For our multi-class classification task, we computed the AUC for each class independently (One-vs-Rest) and reported the macro-average AUC to assess the overall model performance.
